# Case Report: The compound heterozygotes variants in *FLT4* causes autosomal recessive hereditary lymphedema in a Chinese family

**DOI:** 10.3389/fgene.2023.1140406

**Published:** 2023-03-22

**Authors:** Qinqin Xiang, Jing Chen, Xiao Xiao, Bocheng Xu, Hanbing Xie, He Wang, Mei Yang, Shanling Liu

**Affiliations:** ^1^ Department of Obstetrics and Gynecology, West China Second University Hospital, Sichuan University, Chengdu, Sichuan, China; ^2^ Department of Medical Genetics, West China Second University Hospital, Sichuan University, Chengdu, Sichuan, China; ^3^ Key Laboratory of Birth Defects and Related Diseases of Women and Children (Sichuan University), Ministry of Education, Chengdu, Sichuan, China

**Keywords:** *FLT4*, primary lymphedema, autosomal recessive, trio-whole-exome sequencing, case report

## Abstract

**Background:** Lymphedema is a local form of tissue swelling, which is caused by excessive retention of lymph fluid in interstitial compartment caused by impaired lymphatic drainage damage. Primary lymphedema is caused by developmental lymphatic vascular abnormalities. Most cases are inherited as autosomal dominant, with incomplete penetrance and variable expression. Here we report compound heterozygotes variants in *FLT4* of a Chinese family associated with primary lymphedema display autosomal recessive inheritance.

**Case presentation:** Trio-whole-exome sequencing (Trio-WES) was performanced to analyse the underlying genetic cause of a proband with primary lymphedema in a Chinese family. Sanger sequencing was used to validate the variants in proband with primary lymphedema and members of the family with no clinical signs and symptoms. We reported compound heterozygotes for the Fms Related Receptor Tyrosine Kinase 4 (*FLT4*) gene detected in the proband, who carrying two different point variants. One was a missense variant (NM_182925.5; c.1504G>A, p.Glu502Lys), and the other was a recurrent variant (NM_182925.5; c.3323_3325del, p.Phe1108del). The missense variant c.1504G>A was detected in the proband, unaffected father, and unaffected paternal grandmother but not detected in unaffected paternal grandfather. The recurrent variant c.3323_3325del was detected in the proband, unaffected mother, and unaffected maternal grandfather but not detected in unaffected maternal grandmother. Our results suggests the possibility of an autosomal recessive inherited form of primary lymphedema resulting from variants of FLT4 encoding the vascular endothelial growth factor receptor-3.

**Conclusion:** The results of the present study identifed compound heterozygotes *FLT4* variants in a family with primary lymphedema which provides more information for autosomal recessive primary lymphedema caused by *FLT4*.

## Introduction

Lymphedema is a local form of tissue swelling, which is caused by excessive retention of lymph fluid in interstitial compartment caused by impaired lymphatic drainage damage (Brouillard et al., 2014; Grada and Phillips, 2017). Lymphedema is classified as primary or secondary. Primary lymphedema is caused by developmental lymphatic vascular abnormalities. Secondary lymphedema is acquired and caused by disease processes, recurrent infection, trauma or surgery (Kerchner et al., 2008). Primary lymphedema is rare. Due to the difficulty of diagnosis, its epidemiology is not accurate, but the incidence of primary lymphedema in children is predicted to be 1 in 100,000 (Smeltzer et al., 1985; Fitzpatrick, 2008).

Primary lymphedema is caused by anatomic or functional defects in the lymphatic system, resulting in chronic swelling of body parts, and can be an isolated disease or part of a complex syndrome. Most cases are inherited as autosomal dominant, with incomplete penetrance and variable expression. Gene variants can be identified in nearly 30% of patients with primary lymphedema. Primary lymphedema shows a high degree of genetic heterogeneity, more than 20 genes are related to primary lymphedema (Brouillard et al., 2014; Grada and Phillips, 2017). Isolated lymphedema is mainly associated with Fms Related Receptor Tyrosine Kinase 4 (*FLT4*) (also known as vascular endothelial growth factor receptor 3, *VEGFR3*) and vascular endothelial growth factors C (*VEGFC*) (Brouillard et al., 2014).

In this study, genetic analysis of a Chinese family with autosomal recessive primary lymphedema was performed. The Trio-WES analysis revealed that the proband had compound heterozygotes variants for the *FLT4* gene (OMIM:136352; c.1504G>A, p.Glu502Lys and c.3323_3325del, p.Phe1108del).

## Patient and methods

### Patient

**FIGURE 1:**
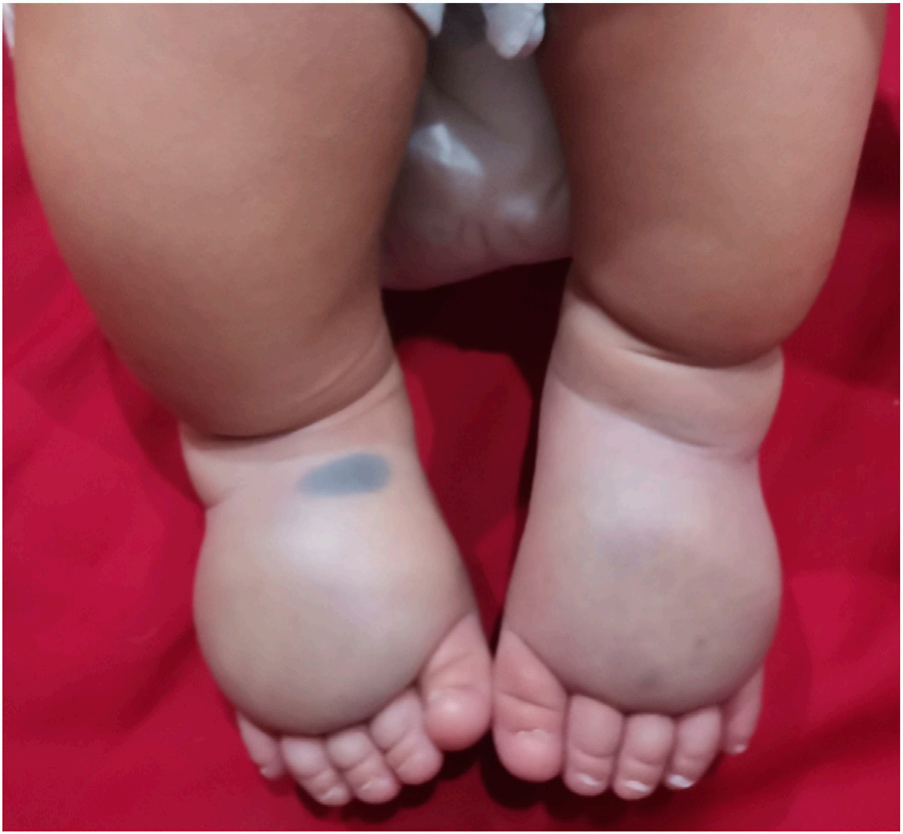
Congenital lymphedema of the lower extremities of the proband.

A Chinese family with primary lymphedema was investigated in this study. The proband was a male infant who present abnormal ultrasound with bilateral lower legs and feet subcutaneous thickening in fetal period. After birth, both lower legs and feet were obviously swollen and show pitting edema (lower leg circumference:left lateral 14.5 cm, right lateral 15 cm; foot circumference:left lateral 12.8 cm, right lateral 14 cm), and ultrasound targeted swelling location indicated that the subcutaneous layer were thickened. The clinical diagnosis was suspicious primary lymphedema (Figure 1; Supplementary Figure S1). The proband’s mother has an adverse pregnancy history, ultrasound in the second trimester showed bilateral lower legs and feet subcutaneous thickening, so the pregnancy was terminated, however, no genetic test was performed with any samples of fetus and no samples were retained. In order to further clarify the diagnosis, Trio-whole-exome sequencing (Trio-WES) was performed to analyse the underlying genetic cause of the family. Suspected variants detected by next-generation sequencing (NGS) were validated by Sanger sequencing.

This study was approved by the Medical Ethics Committee of West China Second University Hospital, Sichuan University, and written informed consent was obtained from all participants.

#### Exome sequencing and validation by sanger sequencing

Total genomic DNA was extracted from the whole blood of the proband, parents, and grandparents using a DNeasy Blood and Tissue DNA kit (Qiagen, Hilden, Germany) according to the manufacturer’s instructions. For detecting the variation carried by the proband, the Nano WES Human Exome V2 (Berry Genomics) was used to capture the sequences. Then the enriched library was sequenced on the Novaseq 6,000 with 150 paired-end reads. The reads were mapped to the human reference genome (hg38) with BWA (v0.7.17). Variant calling was performed by Verita Trekker (v2.1.1). The 1,000 Genomes Project database, and the Genome Aggregation Database (gnomAD, http://gnomad.broadinstitute.org/) were used for minor allele frequency (MAF). For the pathogenicity prediction, CADD (https://cadd.gs.washington.edu), DANN (https://cbcl.ics.uci.edu/public_data/DANN/), dbNSFP (https://sites.google.com/site/jpopgen/dbNSFP), SIFT (http://sift.jcvi.org), PolyPhen-2 (http://genetics.bwh.harvard.edu/pph2), M-CAP (http://bejerano.stanford.edu/mcap/), Mutation Taster (http://www.mutationtaster.org) were used. To select disease-causing variants, we referred to the information from the OMIM (http://www.omim.org), ClinVar (http://www.ncbi.nlm.nih.gov/clinvar) and Human Gene Mutation Database (http://www.hgmd.org). The detailed process for identifying candidate variants is shown in Supplementary Figure S2 and the detailed information of quality control of the proband and parentsis shown in supplemental excel sheet. The 3D structure of the wild-type and mutant *FLT4* homology domains was constructed with SWISS-MODEL (https://swissmodel.expasy.org/).

Sanger sequencing was performed using specific PCR primers designed with Primer Premier 6. The sequences of *FLT4* primers used were *FLT4*-1-F: 5′-AAC​CAC​CTG​CTT​CAG​AAC-3′ and *FLT4*-1-R: 5′-AGA​CAG​ACC​CAG​GAG​AAC-3’; *FLT4*-2-F: 5′-CAT​GTC​AGC​TTC​CTT​GTC​T-3′ and *FLT4*-2-R: 5′-CTT​GCC​TCT​TCT​GGT​CCT-3’. The PCR products were separated and verified by electrophoresis in an 2% agarose gel and characterized by direct Sanger sequencing.

#### Genetic findings

Compound heterozygotes for the *FLT4* gene was detected, the proband carrying two different variants. One was a missense variant (NM_182925.5; c.1504G>A, p.Glu502Lys) derived from his father (II1), and the other was a recurrent variant (NM_182925.5, c.3323_3325del, p.Phe1108del) derived from his mother (II2).

**FIGURE 2:**
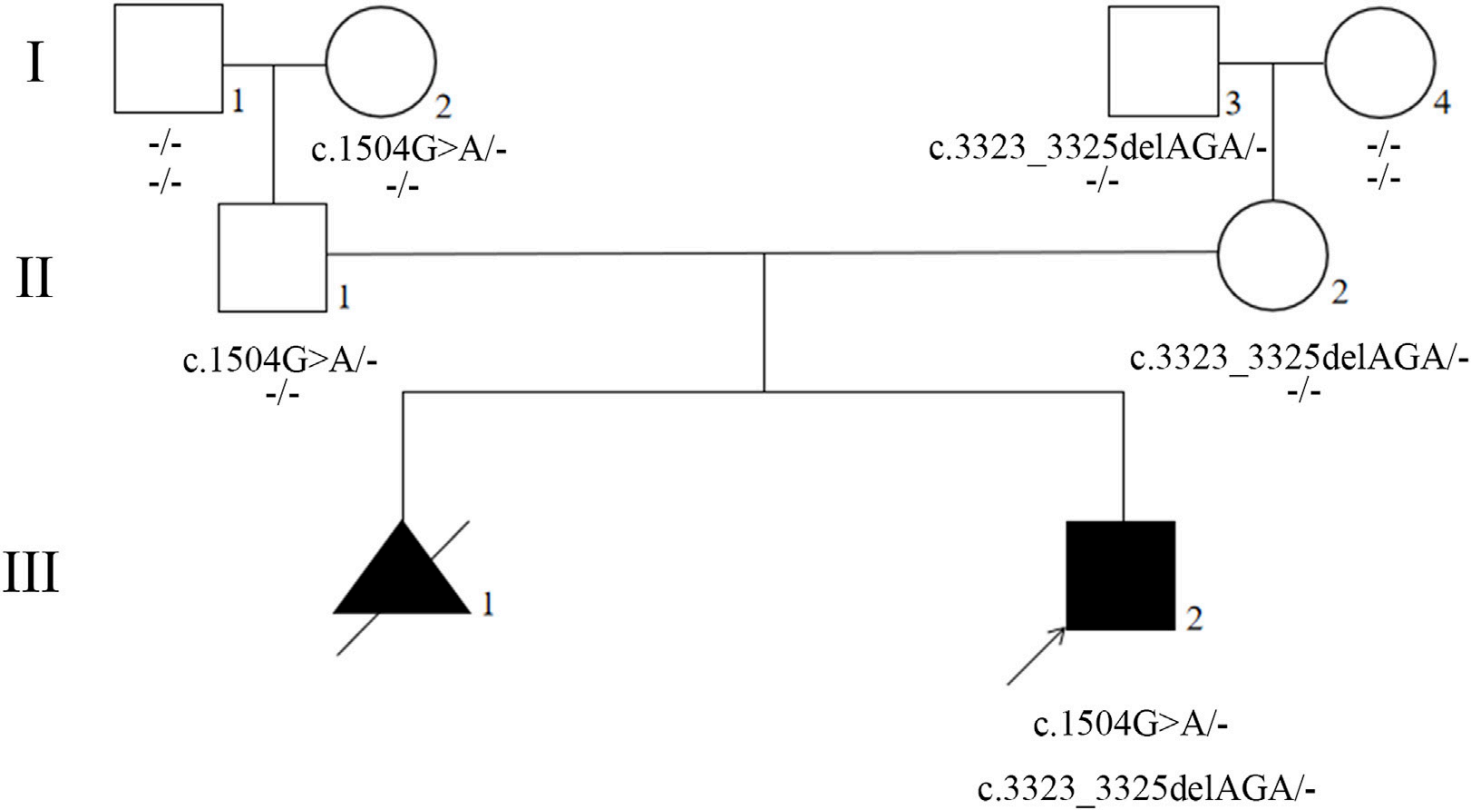
Pedigree of the proband’s family. Generations are shown as I–III. Squares indicate male, and circles indicate female. Empty symbols indicate unaffected individuals and filled symbols indicate affected individuals. Deceased individuals are indicated by a slash (/), the arrow shows the proband.

**FIGURE 3:**
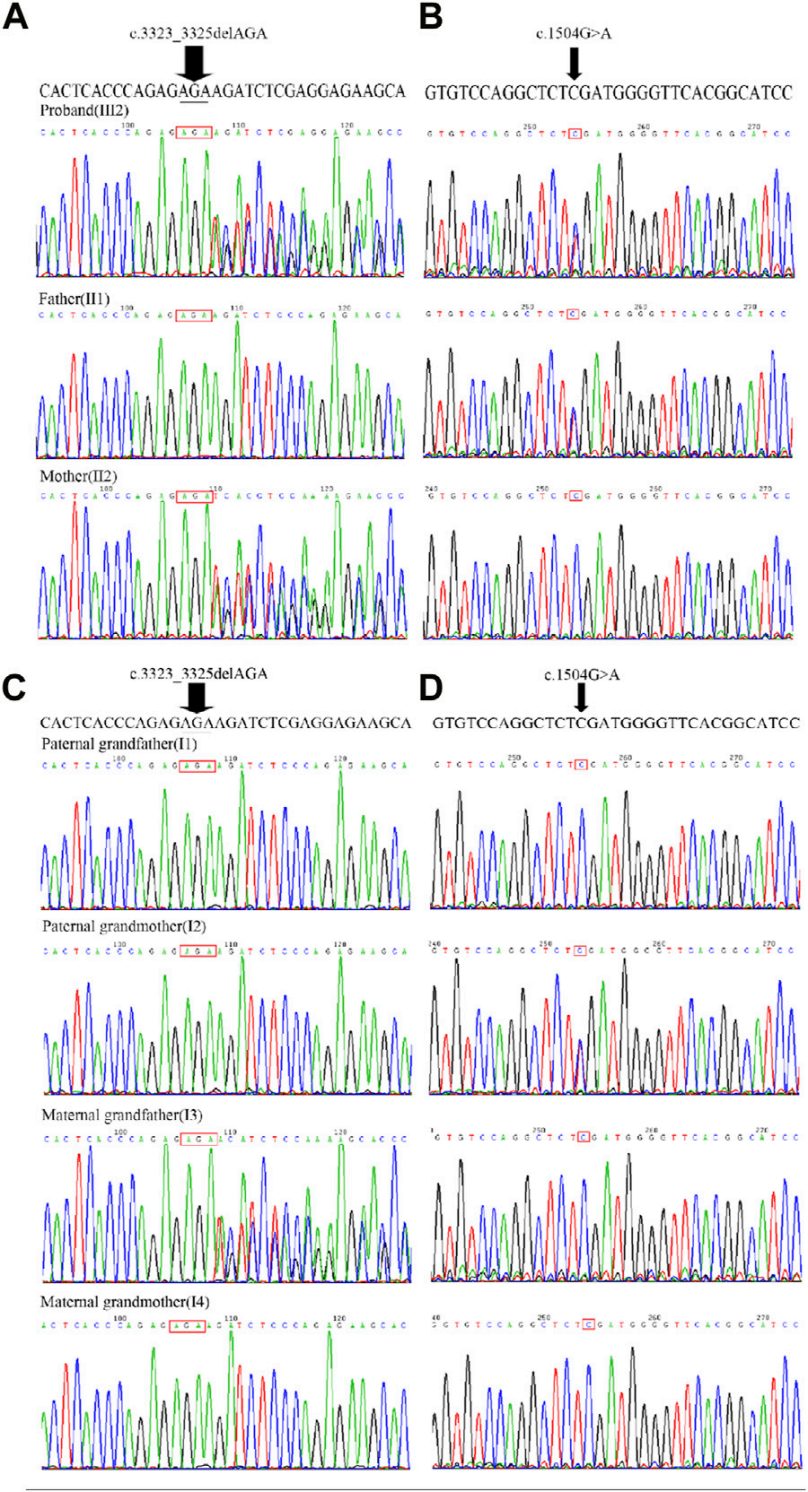
Sanger sequencing chromatograms of FLT4, c.3323_3325del and c.1504G>A. **(A)** The recurrent variant c.3323_3325del (indicated by an arrow) was detected in the proband (III2), unaffected mother (II2), but not detected in unaffected father (II1). **(B)** The missense variant c.1504G>A (indicated by an arrow) was detected in the proband (III2), unaffected father (II1) but not detected in unaffected mother (II2). **(C)** The recurrent variant c.3323_3325del (indicated by an arrow) was detected in the unaffected maternal grandfather (I3) but not detected in unaffected maternal grandmother (I4), unaffected paternal grandmother (I2) and unaffected paternal grandfather (I1). **(D)** The missense variant c.1504G>A was detected in the unaffected paternal grandmother (I2), but not detected in unaffected paternal grandfather (I1), unaffected maternal grandmother (I4)and unaffected maternal grandfather (I3).

The presence of the variants were further validated by Sanger sequencing in proband, parents, and grandparents. The variant c.1504G>A was detected in the proband (III2), unaffected father (II1), and unaffected paternal grandmother (I2) but not detected in unaffected paternal grandfather (I1) (Figures 2, 3; Supplementary Figure S3). The recurrent variant c.3323_3325del was detected in the proband (III2), unaffected mother (II2), and unaffected maternal grandfather (I3) but not detected in unaffected maternal grandmother (I4) (Figures 2, 3; Supplementary Figure S3). These results further support that, in addition to dominantly inherited primary lymphedema, certain FLT4 variants may lead to recessive primary lymphedema.

According to the ACMG Guidelines, neither the proband nor his parents had other pathogenic or likely pathogenic variants about second findings.

#### In silico analysis

**FIGURE 4:**
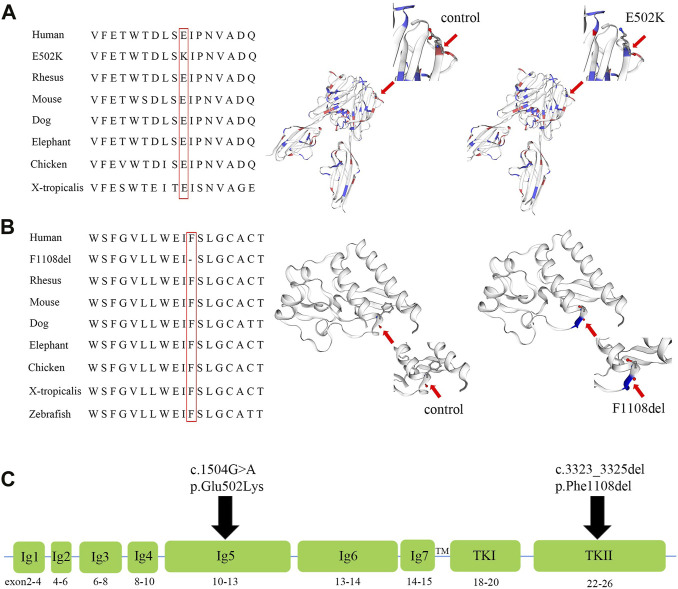
Amino acid alignment and prediction of the protein conformational changes of p.F1108del and p.E502K of the *FLT4* gene, and the diagram of the *FLT4* protein. **(A)** Multiple sequence alignment of the *FLT4* homeodomain from different species of vertebrates and X-tropicalis showing the evolutionary conservation of the glutamic residue at position 502 (highlighted in red box), and models of the wild-type and mutant E502K homeodomains of *FLT4* were constructed with SWISS-MODEL. **(B)** Multiple sequence alignment of the *FLT4* homeodomain from different species of vertebrates,X-tropicalis and Zebrafish showing the evolutionary conservation of the phenylalanine acid at position 1,108 (highlighted in red box), and models of the wild-type and mutant F1108del homeodomains of *FLT4* were constructed with SWISS-MODEL. **(C)** Diagram of the *FLT4* protein showing the functional domains (Ig1 to Ig7 domains and TKI, TKII), and the location of p. E502K and p.F1108del.

The variant c.1504G>A in *FLT4* results in replacement of a glutamic acid (acidic amino acid) by lysine (basic amino acid) at position 502. In silico analysis of the c.1504G>A reveals this substitution may be disease causing, and predicted deleterious by CADD, SIFT, DANN, MetaSVM, MetaLR, and M-CAP. In addition, multiple sequence alignment of the *FLT4* from different species showed the evolutionary conservation of the glutamic acid at position 502 (Figure 4A). The 3D model of wild-type and mutant homologous domains constructed by SWISS-MODEL shows that replacing glutamic acid with lysine at position 502 will not cause a change in the structure, but will cause a change in polarity (Figure 4A).

The variant c.3323_3325del in *FLT4* results in deletion of a phenylalanine at position 1,108. MutationTaster predicted that c.3323_3325del in *FLT4* gene was a disease-causing variant. A change in the same codon that results in p. Phe1108del had been previously reported in patients with primary congenital lymphedema. In addition, multiple sequence alignment of the *FLT4* from different species showed the evolutionary conservation of the glutamic acid at position 1,108 (Figure 4B). The 3D model of wild-type and mutant homologous domains constructed by SWISS-MODEL shows that deletion of a phenylalanine at position 1,108 will cause structure change with destruction of α-helix (Figure 4B).

## Discussion and conclusion

Here we describe the compound heterozygous variants in the *FLT4* gene (NM_182925.5; c.1504G>A, p.Glu502Lys and c.3323_3325del, p.Phe1108del). Our family is the fourth reported to display autosomal recessive inheritance associated with primary lymphedema type I or lymphatic malformation 1 (also known as Milroy disease, OMIM:153100) (Ghalamkarpour et al., 2009; Melikhan-Revzin et al., 2015; Kim and Lim, 2022). Lymphatic malformation 1 is caused by anatomical or functional defects of the lymphatic system, leading to chronic swelling of parts of the body (Gordon et al., 2013a; Balboa-Beltran et al., 2014). The onset is usually at birth or early childhood, but it can also occur later (Gordon et al., 2013a; Balboa-Beltran et al., 2014). Lymphatic malformation 1 is generally due to autosomal dominant variants in the *FLT4* gene, but usually with variable expression and severity (Ferrell et al., 1998; Brouillard et al., 2014; Grada and Phillips, 2017). In previous studies, the autosomal dominant mode of inheritance did not account for all observed familial correlations, suggesting that shared environmental or additional genetic factors may also be important in explaining the observed familial aggregation (Holberg et al., 2001). Three cases of recessive inheritance, caused by variants in the *FLT4* gene, was reported associated with primary lymphedema (Ghalamkarpour et al., 2009; Melikhan-Revzin et al., 2015; Kim and Lim, 2022). In this case, the parents and grandparents with only one variant were phenotypically normal, while the proband with two variants presented with lymphedema. Although there were no samples of fetuses induced by subcutaneous thickening, this adverse pregnancy history also suggests the presence of a family history and suggests the possibility of autosomal recessive inheritance in this family. In general, our research provides more information for autosomal recessive primary lymphedema caused by *FLT4*.

The *FLT4* gene encodes *VEGFR3*, which regulates the development and maintenance of lymphatic system (Monaghan et al., 2021). The *VEGFR3* acts as a cell-surface tyrosine kinase receptor for vascular endothelial growth factors C and D (*VEGFC* and *VEGFD*) (Iljin et al., 2001; Leppanen et al., 2011; Gordon et al., 2013b). When *VEGFC* and *VEGFD* bind to *VEGFR3*, downstream signaling docking sites are produced, regulating the proliferation, migration and survival of lymphatic endothelial cells (LEC) (Lohela et al., 2003). The Chy mouse possesses a heterozygous *FLT4* variant in the tyrosine kinase domain, preventing phosphorylation and resulting in early developmental deficiencies in some lymphatic vessels (Rutkowski et al., 2010). The *FLT4* gene contains 30 exons and encodes seven Ig-homology domains (I-VII), a transmembrane region (TM), and tyrosine kinase domains (TK I and TK II) (Iljin et al., 2001) (Figure 4C). One hundred and forty-three *FLT4* variants have been reported so far worldwide. Most of the variants associated with primary lymphedema were missense variants or single amino acid deletions located in the TK domain of the protein (Evans et al., 2003; Connell et al., 2009; Gordon et al., 2013b; Mendola et al., 2013), while non-sense or frameshift variants in *FLT4* were mainly related to congenital heart defects characterized mainly by tetralogy of Fallot (Jin et al., 2017; Page et al., 2019; Reuter et al., 2019).

The recurrent variant (c.3323_3325del, p. Phe1108del) was identified to be located in the tyrosine kinase II domain (TK II) of the *FLT4* gene (Figure 4C). Evans et al. (2003) identified the same heterozygous variant of *FLT4* in a family with an autosomal dominant form of primary congenital lymphedema, the affected members of this family were variable degrees of painless pitting lower limb edema of congenital onset. In this study, the unaffected members, mother and maternal grandfather, also carried the variant p.Phe1108del, possibly reflecting some residual receptor activity, and suggesting that environmental or other genetic factors may play an important role in the pathogenicity of this variant. In this family, the proband carried another missense variant (c.1504G>A, p.Glu502Lys), which was identified to be located in the Ig-homology V domain (Figure 4C). To date, the variants located in the Ig-homology domains were mainly related to tetralogy of Fallot (Jin et al., 2017; Page et al., 2019), and no disease causing variants in this domain have been reported to be related to primary lymphedema. Combining the *in silico* analysis and the history of this family, we speculate that p.Glu502Lys may be the first reported variant in the Ig-homology domain of VEGFR3 protein that causes primary lymphedema with an autosomal recessive inheritance mode. However, the pathogenicity of these two missense variants needs further functional studies to verify.

Previous molecular analysis of primary lymphedema or Milroy disease was mainly through PCR and direct sequencing of *FLT4* gene encoding the “tyrosine-kinase domain,” or all exons of *FLT4* gene, or screening of a few lymphedema-related genes, which cannot exclude other genetic variants that may explain the clinical presentation of the patients (Kitsiou-Tzeli et al., 2010; Michelini et al., 2012; Michelini et al., 2016). At present, Trio-WES based on next-generation sequencing technology is one of the most effective tools for diagnosis of genetic diseases, which can detect exons of all known gene. Therefore, our results provide accurate genetic information for targeted treatment, genetic counseling and subsequent prenatal diagnosis of the patients in this family.

In conclusion, we successfully identified compound heterozygous variants for the *FLT4* gene (OMIM:136352; c.1504G>A, p.Glu502Lys and c.3323_3325del, p.Phe1108del) in this family. The missense variant p.Glu502Lys is the first reported disease-causing variant in the Ig-homology domain of *VEGFR3* protein that causes primary lymphedema. Furthermore, our study provides more information for autosomal recessive primary lymphedema caused by *FLT4*.

## Data Availability

The datasets for this article are not publicly available due to concerns regarding participant/patient anonymity. Requests to access the datasets should be directed to the corresponding authors.

## Abbreviations

*FLT4*, Fms Related Receptor Tyrosine Kinase 4; VEGFR3, vascular endothelial growth factor receptor 3; VEGFC, vascular endothelial growth factors C; Trio-WES, Trio-whole-exome sequencing; NGS, next-generation sequencing; VEGFD, vascular endothelial growth factors D; LEC, lymphatic endothelial cells; TM, transmembrane region; TK, tyrosine kinase.
