# Supplementary figures and images for: Case Report: The compound heterozygotes variants in FLT4 causes autosomal recessive hereditary lymphedema in a Chinese family
Source: Front Genet. 2023 Mar 22;14:1140406. doi: 10.3389/fgene.2023.1140406 (PMC10073681; doi:10.3389/fgene.2023.1140406)

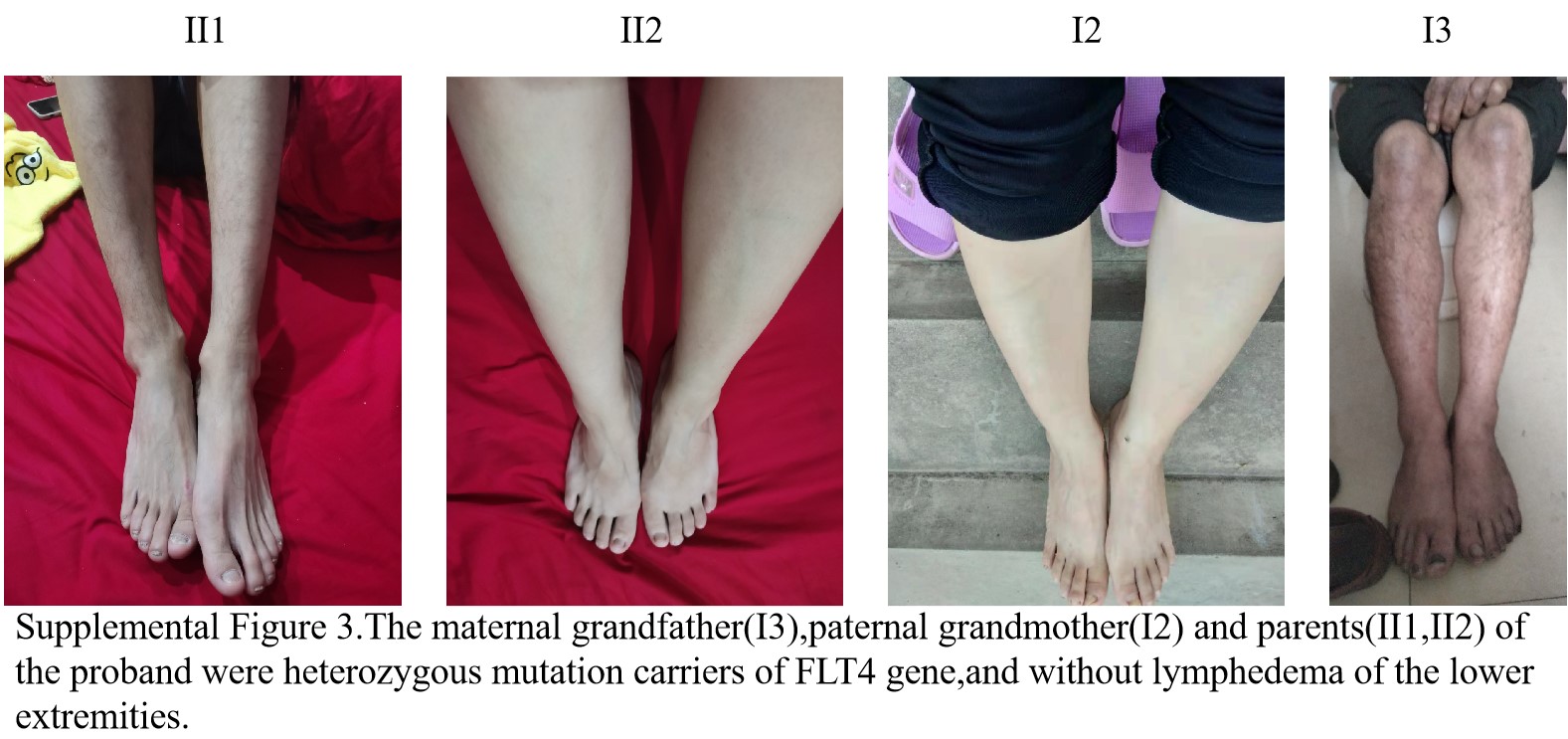

Supplement: Supplementary file 1 [file Image3.JPEG]

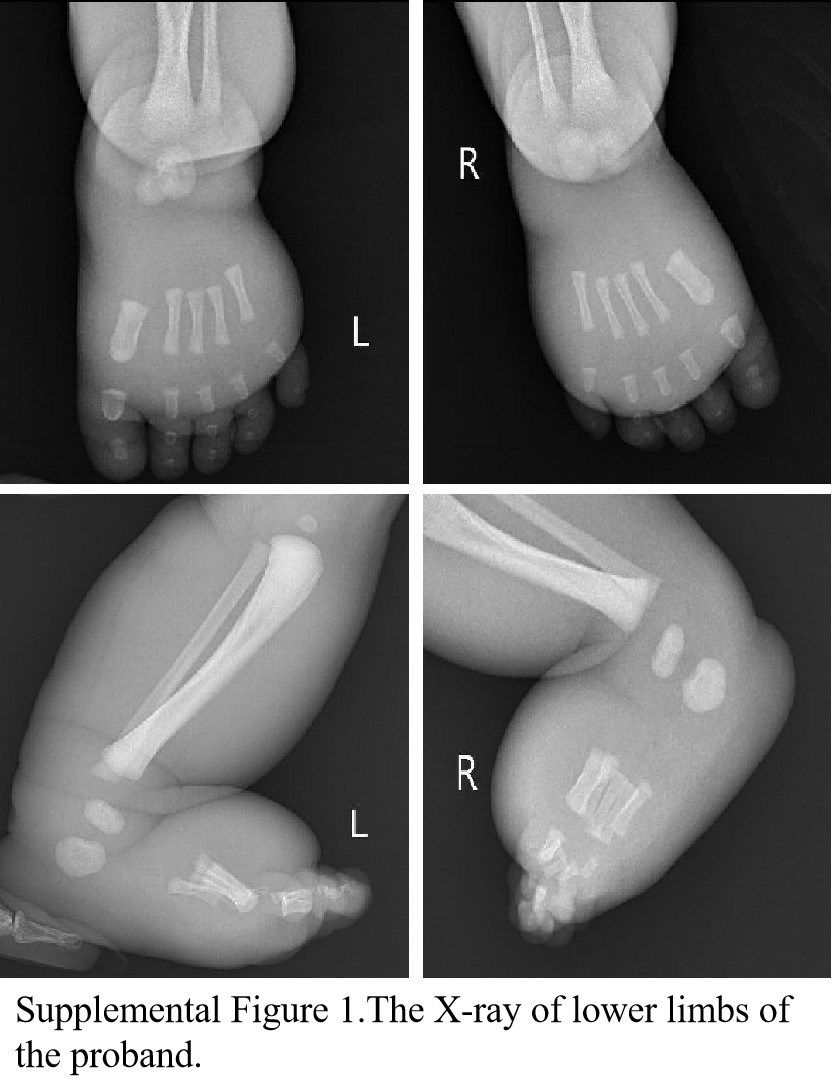

Supplement: Supplementary file 2 [file Image1.JPEG]

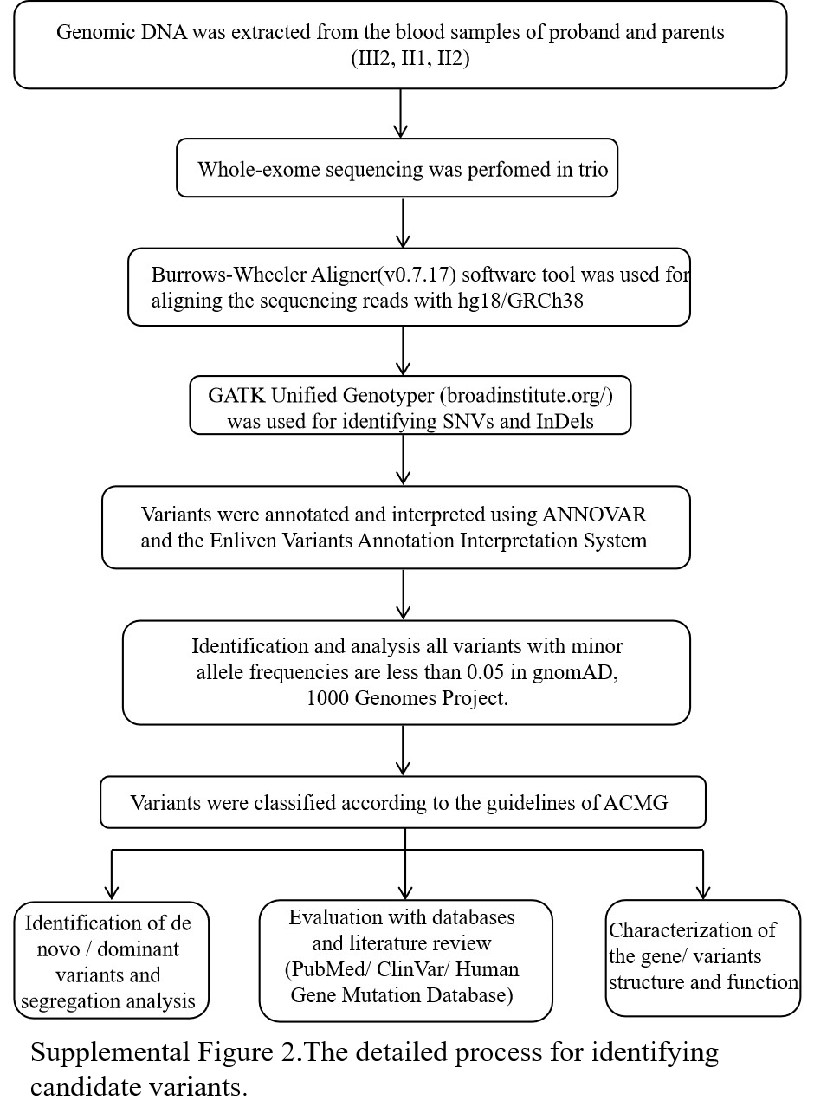

Supplement: Supplementary file 3 [file Image2.JPEG]
